# Conjugated Mesopolymer Achieving 15% Efficiency Single‐Junction Organic Solar Cells

**DOI:** 10.1002/advs.202105430

**Published:** 2022-01-22

**Authors:** Bing Zheng, Jianling Ni, Shaman Li, Yuchen Yue, Jingxia Wang, Jianqi Zhang, Yongfang Li, Lijun Huo

**Affiliations:** ^1^ School of Chemistry Beihang University Beijing 100191 P. R. China; ^2^ Beijing National Laboratory for Molecular Sciences CAS Key Laboratory of Organic Solids Institute of Chemistry Chinese Academy of Sciences Beijing 100190 China; ^3^ School of Chemical Science University of Chinese Academy of Sciences Beijing 100049 China; ^4^ Key Laboratory of Bioinspired Smart Interfacial Science Technical Institute of Physics and Chemistry Chinese Academy of Sciences Beijing 100190 P. R. China; ^5^ School of Future Technology University of Chinese Academy of Sciences (UCAS) Beijing 100049 P. R. China; ^6^ CAS key laboratory of nanosystem and hierarchical fabrication CAS Center for Excellence in Nanoscience National Center for Nanoscience and Technology Beijing 100049 P. R. China

**Keywords:** benzodifuran, mesopolymer, molecular weight, organic solar cell, power conversion efficiency

## Abstract

The high‐performance organic solar cells (OSCs) tend to choose the polymers with high molecular weight as donors, which easily produce good crystallinity to facilitate intermolecular charge transfer. However, these polymers usually accompanied by the low solubility and synthetic difficulty, increasing batch‐to‐batch variations. The proposal of conjugated mesopolymers (molar mass (*M*_n_) in 1–10 kDa) can overcome these problems. Herein, a new mesopolymer, MePBDFCl_*H*_ as donor material is designed and synthesized, and firstly applied in OSCs. As a comparison, other lower molecular weight mesopolymer of MePBDFCl_*L*_ and higher molecular weight polymer of PBDFCl with same structure are also prepared and investigated. Because of its appropriate phase separation and miscibility in the blend film, the MePBDFCl_*H*_ exhibits the highest power conversion efficiency (PCE) of 15.06% among the three materials. Meanwhile, the champion PCE is a new record for benzo[1,2‐b:4,5‐b′]difuran‐based photovoltaic materials. Importantly, comparing to the pronounced PCE decrease of polymer PBDFCl by about 12%, a slightly PCE difference for mespolymer MePBDFCl_*L*_ is only less than 5%, reducing the batch‐to‐batch variation. This work not only suggests that the benzo[1,2‐b:4,5‐b′]difuran unit is a promising electron‐donating core but also shows that the mesopolymers have great potentials to produce the low‐differentiated and high‐performance organic photovoltaic materials.

## Graphical abstract

Conjugated mesopolymer is first applied in non‐fullerene solar cells. The champion device based on MePBDFCl_*H*_/Y6 shows a power conversion efficiency (PCE) of 15.06%, which is attributed to the efficient intermolecular charge transfer. Meanwhile, the champion PCE is a new record for benzo[1,2‐b:4,5‐b′]difuran‐based organic solar cells. More importantly, the batch‐to‐batch variation is effectively reduced by adopting mesopolymer.

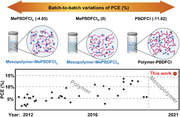

## 1 Introduction

Solution‐processed bulk heterojunction organic solar cells (OSCs) have made considerable development due to their unique merits of low‐cost, lightweight, flexible, and simple device processing in recent decades.^[^ ^1^, ^2^, ^3^, ^4^, ^5^ ^]^ With the innovation of photovoltaic materials containing polymer donor and small molecule acceptor, the photovoltaic performance of polymer solar cells (PSCs) has obtained significant improvement. In particular, the emergence of non‐fullerene acceptors has realized high power conversion efficiency (PCE) over 18%, attaining the preliminary level of commercialization.^[^ ^6^, ^7^, ^8^, ^9^, ^10^, ^11^, ^12^, ^13^ ^]^

**Figure 1:**
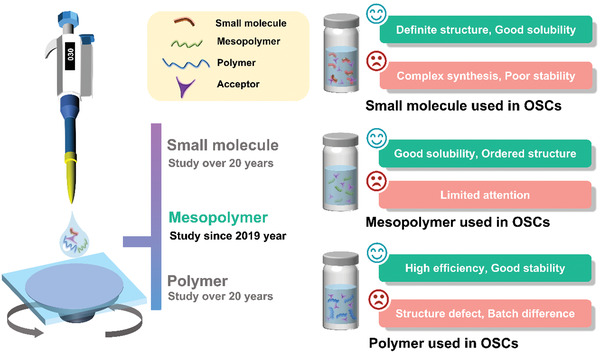
The characteristics of small molecule, mesopolymer, and polymer for the application in organic photovoltaics.

Indeed, to pursue high PCE, for the variously excellent non‐fullerene acceptors such as 3,9‐bis(2‐methylene‐(3‐(1,1‐dicyanomethylene)‐indanone))‐5,5,11,11‐tetrakis(4‐hexylphenyl)‐dithieno[2,3‐d:2′,3′‐d′]‐s‐indaceno[1,2‐b:5,6‐b′]dithiophene (ITIC) and (2,2′‐((2Z,2′Z)‐((12,13‐bis(2‐ethylhexyl)‐3,9‐diundecyl‐12,13‐dihydro‐[1,2,5]thiadiazolo[3,4‐e] thieno[2″,3″:4′,5′]thieno[2′,3′:4,5]pyrrolo[3,2‐g]thieno[2′,3′:4,5] thieno[3,2‐b]indole‐2,10‐diyl)bis(methanylylidene)) bis(5,6‐difluoro‐3‐oxo‐2,3‐dihydro‐1H‐indene‐2,1‐diylidene)) dimalononitrile) (Y6), et al., a reasonable match in absorption region, energy levels, and ideal morphology between acceptor and donor is necessary.^[^ ^14^, ^15^, ^16^, ^17^, ^18^ ^]^ Despite these factors, strong crystalline polymer donor featuring high molecular weight also is one indispensable factor, since high molecular weight commonly accompanies by good crystallinity which facilitates intermolecular charge transfer.^[^ ^19^, ^20^, ^21^, ^22^, ^23^, ^24^, ^25^, ^26^, ^27^ ^]^ However, this high molecular weight requirement in polymer not only lead to the low solubility but also cause difficulty in synthesis, especially, controlling the appropriate high molecular weight region. Although the adoption of the modified polymerization conditions such as mixed solvents of toluene and N,N‐dimethylformamide (DMF), or combined catalyst could realize high molecular weight, an inevitable negative effect appears in obvious batch‐to‐batch variations.^[^ ^28^, ^29^, ^30^, ^31^ ^]^ Taking two best‐known polymer donors as examples, (poly([2,6′‐4,8‐di(5‐ethylhexylthienyl)benzo[1,2‐b;3,3‐b]dithiophene]{3‐fluoro‐2[(2‐ethylhexyl)carbonyl]thieno[3,4‐b]thiophenediyl}))‐Th,(poly[(2,6‐(4,8‐bis(5‐(2‐ethylhexylthio)‐4‐fluorothiophen‐2‐yl)‐benzo[1,2‐b:4,5‐b′]dithiophene))‐alt‐(5,5‐(1′,3′‐di‐2‐thienyl‐5′,7′‐bis(2‐ethylhexyl)benzo[1′,2′‐c:4′,5′‐c′]dithiophene‐4,8‐dione)]) (PM6)[32] and poly[(2,6‐(4,8‐bis(5‐(2‐ethylhexyl‐3‐fluoro)thiophen‐2‐yl)‐benzo[1,2‐b:4,5‐b′]dithiophene))‐alt‐(2‐butyloctyl) thiophen‐2‐yl)‐8‐(4‐(2‐butyloctyl)‐5‐methylthiophen‐2‐yl)dithieno[3′,2′:3,4;2″,3″:5,6]benzo[1,2‐c][1,2,5]thiadiazole)]) (D18).^[^ ^8^ ^]^ Some reports have showed that PM6 with high molecular weight can produce high PCE of over 17%.^[^ ^33^, ^34^, ^35^, ^36^, ^37^, ^38^ ^]^ But the efficient PM6 batch must react in a mixed solvents of toluene and DMF system, which is difficult to control in molecular weight consistency. As far as D18, only in a narrow number‐averaged molecular weight (Mn) range about 70 kDa can exhibit relatively high efficiency for corresponding PSC device.^[^ ^39^ ^]^ Under these circumstances, the incapable volume‐produce of high‐efficiency polymer donors with high molecular weight becomes one of archcriminal for impeding the commercialization of OSCs. Recently, the proposal of conjugated mesopolymers (molar mass (Mn) between 1 and 10 kDa) have been proved to be an effective strategy for overcoming the low solubility and batch‐to‐batch variations.^[^ ^40^, ^41^ ^]^ The mesopolymers possess facile synthesis, high molecular regularity, and good solution processability.^[^ ^42^, ^43^ ^]^ Meanwhile, the appropriate molecular weights for mesopolymers can be easily obtained by controlling the reaction time or adopting the different polymerization process. However, the mesopolymers as donor materials are infrequent in organic photovoltaic field (**Figure** **1**). Hence, designing and synthesizing a mesopolymer donor with high efficiency is an interesting research topic.

Herein, in this contribution, we reported a new mesopolymer donor, named (poly[(2,6‐(4,8‐bis(5‐(2‐ethylhexyl‐4‐chloro‐2‐thienyl)‐benzo[1,2‐b:4,5‐b′]difuran))‐alt‐(2‐butyloctyl)thiophen‐2‐yl)‐8‐(4‐(2‐butyloctyl)‐5‐methylthiophen‐2 yl)dithieno[3′,2′:3,4;2′′,3′′:5,6]benzo[1,2‐c][1,2,5]thiadiazole)]) (MePBDFCl). This mesopolymer adopted benzo[1,2‐b:4,5‐b′]difuran (BDF) as electron‐donating core since the oxygen atomic radius (1.52 Å) of furan is smaller than the sulfur atomic radius (1.80 Å), which easily formed intermolecular stacking. Meanwhile, the furan‐based photovoltaic materials hold some unique fascinations in comparison with thiophene‐based materials, such as, good solubility, strong fluorescence, abundant product from renewable resources, high mobility, and so on.^[^ ^44^, ^45^, ^46^, ^47^, ^48^, ^49^, ^50^ ^]^ To precisely evaluate the difference between mesopolymer and polymer on the photovoltaic performance, two contrasts, a mesopolymer MePBDFCl*_L_* with lower molecular weight and a polymer PBDFCl with higher molecular weights were also synthesized. In the meantime, the non‐fullerene Y6 was used as acceptor in the PSCs, to match the absorption and energy levels of polymer. As the *M*_n_ increases, three materials exhibited different molecular packing and photovoltaic properties. To elucidate the origin of the differences in the solar cell performance, the molecular weight dependence of the charge carrier mobility, crystallization behavior and molecular packing, film morphology, and the miscibility in the blends were investigated. As a result, the medium molecular weight of MePBDFCl*_H_* achieved the highest PCE value of 15.06%, which is the best PCE for BDF‐based polymers and the highest efficiency record for furan based photovoltaic materials. Meanwhile, this work is the first time to report mesopolymer application in organic photovoltaics. It was worth noting that the different PCEs between MePBDFCl*_L_* and MePBDFCl*_H_* was less than 5% under the optimized photovoltaic device conditions. In contrast, the polymer PBDFCl with high molecular weight exhibited a pronounced drop in PCE by 12%, which indicated that the proposed mesopolymer was an effective strategy to reduce the batch‐to‐batch variation. This successful case not only proves that the BDF unit is a promising electron‐donating core for OSCs, but also indicates that the mesopolymer possessed more potentials for the application in the organic photovoltaics.

## 2 Results and Discussion

### 2.1 Synthesis and Characterization

**Figure 2:**
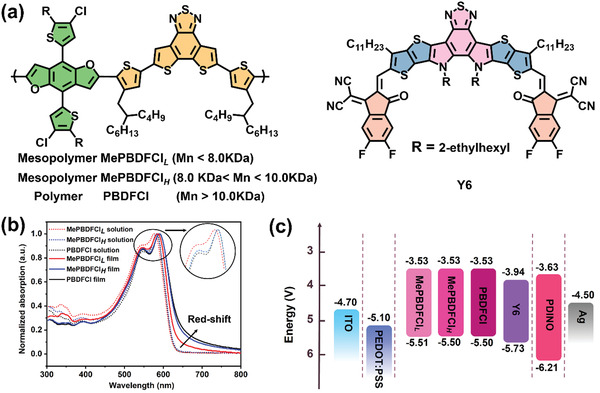
a) Molecular structures of MePBDFCl*_L_*, MePBDFCl*_H_*, PBDFCl, and Y6. b) The normalized UV–vis absorption spectra of polymers in solution and films. c) Energy level diagram of the related materials used in OSCs devices.

The molecular structures of PBDFCl and the acceptor Y6 are showed in **Figure** **2**a, and corresponding synthetic routes are summarized in Scheme S1, Supporting Information. To acquire materials with different molecular weights, reaction conditions of the polymerization are carefully tuned by changing reaction time and Pd catalyst, giving two mesopolymers (MePBDFCl*_L_* and MePBDFCl*_H_*) and a polymer PBDFCl. All of them possess adequately good solubility in chloroform (CHCl_3_) and chlorobenzene at room temperature. The molecular weights (*M*_n_) of MePBDFCl*_L_*, MePBDFCl*_H_*, and PBDFCl are 8.0, 9.9, and 17.1 kDa with appropriate polydispersity index (PDI) of 1.51, 1.41, and 1.60, respectively. The results show that MePBDFCl*_L_* and MePBDFCl*_H_* belong to mesopolymers due to their low molecular weights between 1 and 10 kDa. Meanwhile, all these donor materials have good thermostability with a similar decomposition temperature beyond 370 °C under nitrogen atmosphere. (Figure S1, Supporting Information).

The UV–vis spectroscopy was used to investigate the optical properties for these mesopolymers and the polymer in dilute chloroform solution and films. As shown in Figure 2b, MePBDFCl*_H_* and PBDFCl exhibited the similar main absorption peaks located at ≈548 nm and the strong aggregated shoulder peaks at ≈583 nm in dilute solution, which were distinct red‐shift compared to those of MePBDFCl*_L_*. In thin films, MePBDFCl*_L_*, MePBDFCl*_H_*, and PBDFCl displayed the different shoulder peaks and onset absorption edge. These results suggested that the MePBDFCl*_L_* with low molecular weight had relatively weak aggregation in comparison with MePBDFCl*_H_* and PBDFCl. Meanwhile, MePBDFCl*_H_* exhibited the comparable aggregation behavior with the polymer PBDFCl whatever in dilute solution or films. Therefore, the mesopolymer with appropriate molecular weight can potentially undertake the efficient molecular stacking in OSCs. Due to the stacking difference, the optical bandgaps are 1.96, 1.94, and 1.93 for MePBDFCl*_L_*, MePBDFCl*_H_*, and PBDFCl, respectively. The detailed data of the optical properties are summarized in **Table** **1**. The highest occupied molecular orbital (HOMO) and the lowest unoccupied molecular orbital (LUMO) are determined by the cyclic voltammetry (CV) to evaluate the electrochemical property of three materials. The Figure S2a, Supporting Information, shows that the onset oxidation and reduction potentials, and corresponding *E*_HOMO_/*E*_LUMO_ values of MePBDFCl*_H_* could be calculated according to the equations of *E*_HOMO_/*E*_LUMO_ = −e (*E*_ox/red_ + 4.71) (eV). The measured HOMO/LUMO levels were −5.51 eV/−3.53 eV for PBDFCl, and these levels of the mesopolymers were almost comparable with PBDFCl. The energy level diagrams of donor and acceptor display a clear comparison in Figure S2b, Supporting Information, indicating effective exciton dissociation in their interfaces.

**Table 1:** The summary of optical and electronic properties for the mesopolymers and the polymer

Polymer	*λ*_max_ [nm] solution film		*λ*_onset_ ^a)^ [nm]	*E*_g_^opt^ ^b)^ [eV]	HOMO [eV]	LUMO [eV]	*E*_g_^ec^ ^c)^ [eV]	*M*_n_	*M*_w_	PDI
MePBDFCl*_L_*	544	548	632	1.96	−5.51	−3.53	1.98	8.0 k	12.1 k	1.51
MePBDFCl*_H_*	550	548	639	1.94	−5.50	−3.53	1.97	9.9 k	14.0 k	1.41
PBDFCl	550	548	641	1.93	−5.50	−3.53	1.97	17.1 k	27.3 k	1.60

^a)^ Absorption edge of the polymer films;

^b)^ Calculated from the absorption edge of the polymer films: *E*_g_^opt^ = 1240/*λ*_edge_;

^c)^ *E*_g_^ec^ = |*E*_LUMO_ − *E*_HOMO_|.

**Figure 3:**
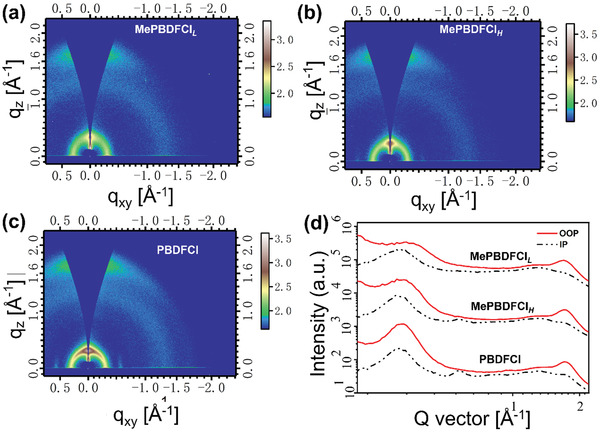
The GIWAXS images of mesopolymers of a) MePBDFCl*_L_*, b) MePBDFCl*_H_*, and the polymer of c) PBDFCl neat films, respectively. d) The corresponding IP and OOP line cuts.

To investigate the effect of different molecular weights of mesopolymer and polymer on the molecular packing and crystalline features in the thin film, the grazing incidence wide angle X‐ray scattering (GIWAXS) measurement was adopted. The 2D GIWAXS profiles and corresponding line‐cuts of in‐plane (IP) and out‐of‐plane (OOP) direction are exhibited in **Figure** **3**. First, three materials with different molecular weights displayed uniform face‐on molecular orientation and distinct *π*‐*π* stacking diffraction peaks in the OOP direction. The location of lamellar (100) peak and *π*–*π* stacking peak (010) changed with the increasement of molecular weights. On the one hand, the lamellar (100) peaks were observed at *q* ≈ 0.317, 0.310, and 0.308 Å^–1^ for MePBDFCl*_L_*, MePBDFCl*_H_*, and PBDFCl, respectively, corresponding to a packing distance of 19.81, 20.26, and 20.39 Å. On the other hand, the MePBDFCl*_L_*, MePBDFCl*_H_*, and PBDFCl showed the *π*–*π* stacking peak located at *q* ≈ 1.702, 1.713, and 1.734 Å^–1^ in the OOP direction, corresponding to the *π*–*π* stacking distance of 3.69, 3.67, and 3.62 Å, respectively. The results suggested that the crystallinity could increase as the improvement of molecular weights. Meanwhile, due to the smaller atom radius of oxygen in furan than sulfur atom in thiophene unit, both the mesopolymers of MePBDFCl*_L_* and MePBDFCl*_H_* are easy to form efficient molecular *π*‐*π* stacking whether the low or high molecular weight. That is to say, the adoption of BDF as monomer to finely construct reasonable low molecular weight mesopolymeric region (8 KDa< *M*_n_ <10 KDa), the slightly decreased *π*–*π* stacking degree has not affect its basic strong crystalline properties, which could facilitate efficient charge transportation.

### 2.2 Photovoltaic Properties

**Figure 4:**
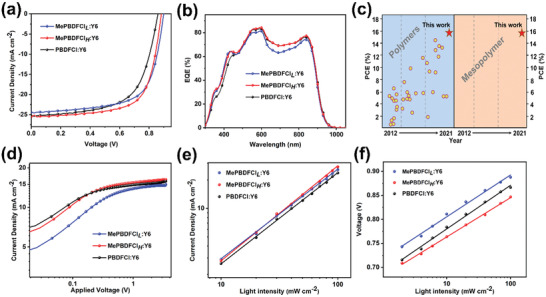
a) *J*–*V* curves of the best OSCs based on materials: Y6 under the illumination of AM1.5G, 100 mW cm^−2^. b) EQE curves of the corresponding OSCs. c) PCE values based on BDF‐based polymer and mesopolymers as donors reported since 2012 year. d) *J*_ph_ versus *V*_eff_ of the OSCs based on materials: Y6. e) Light intensity dependence of *J*_sc_ values of the corresponding OSCs. f) Light intensity dependence of *V*_oc_ values of the corresponding OSCs.

The photovoltaic performance of these two mesopolymers and the polymer with different molecular weights was evaluated via using a device configuration of ITO/poly(3,4‐ethylenedioxythiophene):poly(styrenesulfonate)/polymers:Y6 /PDINO/Ag. (Figure 2d) The detailed optimization of devices including the various D/A ratio, the additive content, and annealing temperature are shown in Figure S3 and Tables S1–S3, Supporting Information. The optimized device fabrication conditions were different for the three materials. Thereinto, the main difference was that MePBDFCl*_L_* needed 0.5% chloronaphthalene (CN) as additive to obtain an optimal condition, inversely, MePBDFCl*_H_* and PBDFCl‐based devices only needed thermal annealing without additive. The current density–voltage (*J*–*V*) curves of the optimized devices based on MePBDFCl*_L_*:Y6, MePBDFCl*_H_*:Y6, and PBDFCl:Y6 are displayed in **Figure** **4**a, and detailed parameters are listed in **Table** **2**. Among the three materials, the MePBDFCl*_H_*‐based OSCs exhibited the highest PCE of 15.06%, with *V*_oc_ of 0.883 V, *J*_sc_ of 24.96 mA cm^−2^, and FF of 68.32%. For the other polymers, the optimal OSCs showed PCEs of 14.36% and 13.28% for MePBDFCl*_L_*‐ and PBDFCl‐based devices, respectively. It is worth noting that the variations of the optimized PCEs are less than 5% between the mescopolymers of MePBDFCl*_L_* and MePBDFCl*_H_*, which is far less than that of the polymer of PBDFCl (≈12% discount). The result indicates that the proposal of mesopolymers is an effective strategy to reduce the batch‐to‐batch variation. Additionally, in spite of little difference of energy levels among the three materials, the diversity of molecular aggregation and phase separation with the varied molecular weights can caused their different *V*_oc_ values. On the one hand, the *V*_oc_ is related with energy levels of materials; on the other hand, the microstructure of blend film plays an important role in affecting the cell voltages. The difference of miscibility among the three donor materials with the acceptor could form diverse microstructures, influencing the carrier lifetimes and recombination losses, thus achieving the discrepant *V*_oc_.^[^ ^51^, ^52^, ^53^ ^]^ Figure 4b shows the external quantum efficiency spectra of the optimal devices. All of devices displayed strong photoresponse in the wavelength range of ≈420–880 nm. Compared to MePBDFCl*_L_* and PBDFCl, the MePBDFCl*_H_*‐based OSC exhibited largest photoresponse area, thereby generating the highest *J*_sc_ value due to the more efficient photon‐harvesting. On the contrary, the OSC based MePBDFCl*_L_*:Y6 had the least photoresponse area, thus yielding the lowest *J*_sc_ value. It's worth mentioning that the mesopolymer is first used in organic photovoltaics and the champion PCE of 15.05% is a new record for BDF‐based PSCs (Figure 4c). The corresponding photovoltaic properties of the BDF‐based materials in recent years are summarized in Table S4, Supporting Information.

**Table 2:** Summary of device parameters of polymers: Y6‐based devices based on optimized conditions under the illumination of AM 1.5 G, 100 mW cm^–2^

Polymers:Y6	*V*_oc_ [V]	*J*_sc_ [mA cm^−2^]	*J*_sc cal_ [mA cm^−2^]^c)^	FF [%]	PCE [%]^d)^
MePBDFCl*_L_*:Y6^a)^	0.90 (±0.004)	23.88 (±0.24)	22.98 (±0.25)	66.87 (±0.005)	14.36 (±0.017)
MePBDFCl*_H_*:Y6^b)^	0.88 (±0.004)	24.96 (±0.25)	24.12 (±0.25)	68.32 (±0.005)	15.06 (±0.017)
PBDFCl:Y6^b)^	0.86 (±0.003)	24.70 (±0.25)	23.80 (±0.25)	62.52 (±0.005)	13.28 (±0.016)

^a)^ Fabricated with 0.5% CN and annealing at 110 °C;

^b)^ Fabricated with annealing at 110 °C;

^c)^ Integrated from EQE values;

^d)^ All average values were calculated from ten devices.

In addition, the charge carrier mobilities of the neat and blend films for three materials were also estimated by the space‐charge‐limited current method, and corresponding parameters are summarized in Figure S4 and Table S5, Supporting Information. The results showed that the hole motility (*μ*_h_) values were 3.49 × 10^−4^, 4.78 × 10^−4^, and 4.46 × 10^−4^ cm^2^ V^−1^ s^−1^ for MePBDFCl*_L_*, MePBDFCl*_H_*, and PBDFCl neat films, respectively. The MePBDFCl*_H_* with a middle molecular‐weight has slightly improved charge mobility than PBDFCl with high molecular‐weight, which signifies the middle molecular‐weight has been enough to generate continuous ordered domains, serving as pathways and therefore benefit efficient charge transportation.^[^ ^54^, ^55^, ^56^ ^]^ On the other hand, Previous research has demonstrated that polydispersity of polymer is important for achieving high carrier mobilities. Even doping of small amounts of low molecular weight material can also limit interchain hopping, reducing the charge carrier mobility.^[^ ^57^ ^]^ The material purity of MePBDFCl*_H_* is higher than that of PBDFCl according to the PDI data, therefore, the deficiency of length of the material is counteracted by crystalline purity for MePBDFCl*_H_*. For the blend films, the MePBDFCl*_L_*, MePBDFCl*_H_*, and PBDFCl exhibited the hole mobility (*μ*_h_)/electron mobility (*µ*_e_) of 3.17 × 10^−4^/3.56 × 10^−4^ cm^2^ V^−1^ s^−1^, 4.57 × 10^−4^/4.84 × 10^−4^ cm^2^ V^−1^ s^−1^, as well as, 3.73 × 10^−4^/4.45 × 10^−4^ cm^2^ V^−1^ s^−1^, respectively. The ratios of hole and electron mobility in binary blend film were obtained: 0.89 for MePBDFCl*_L_*, 0.94 for MePBDFCl*_H_*, and 0.84 for PBDFCl, respectively. It is well known that the more balanced hole/electron mobilities ratio can boost the FF. Consequently, the most balanced *μ*_h_/*µ*_e_ ratio coincided with the highest FF for OSCs based on MePBDFCl*_H_*:Y6 among these devices. On the other hand, although the *μ*_h_/*µ*_e_ ratio of MePBDFCl*_L_*:Y6 was higher than that of PBDFCl blends, the lower charge mobility resulted in lower *J*_sc_ for MePBDFCl*_L_*‐based device.

To further gain a better understanding of the mesopolymers and the polymer on the exciton dissociation and charge recombination, the dependency of photocurrent density (*J*_ph_) versus the effective voltage (*V*_eff_), as well as, the relationship between *J*_sc_/*V*_oc_ and light intensity (*P*_light_) were measured. Generally, *J*_ph_ equals to *J*_L_–*J*_D_, where *J*_L_ and *J*_D_ represent the current density under illumination and dark, respectively. *V*_eff_ equals *V*_0_–*V*, where *V*_0_ and *V* are the voltage when *J*_ph_ = 0 and the applied voltage, respectively. When *V*_eff_ ≥ 2 V, the *J*_ph_ reaches saturation (*J*_sat_), consequently, the probability of charge dissociation and collection of photocurrent carriers *P* (E, T) can be estimated by the ratio of *J*_ph_/*J*_sat_.^[^ ^58^ ^]^ As shown in Figure 4d, the values of *P* (E, T) for the OSCs based on MePBDFCl*_L_*:Y6, MePBDFCl*_H_*:Y6, and PBDFCl:Y6 are 95.4%, 97.8%, and 96.3%, respectively. The result signified that the MePBDFCl*_H_* based devices possessed more efficient exciton dissociation and charge extraction than those of MePBDFCl*_L_* and PBDFCl based devices. In addition, the charge‐recombination mechanism was investigated by studying the relationship *J*_sc_ and *P*_light_ according to the equation *J*_sc_∝*P*_light_^S^.^[^ ^59^ ^]^ The value of *S* closer to 1 means less charge‐recombination. Figure 4e exhibits the *S* values for the MePBDFCl*_L_*, MePBDFCl*_H_*, and PBDFCl‐based devices, which were 96.3%, 98.9%, and 97.2%, respectively, indicating the less charge‐recombination in the MePBDFCl*_H_*‐based devices. Furthermore, the dependency between *V*_oc_ and *P*_light_ can contribute to confirming the mode of charge‐recombination. When the slope of fitted data equals *k*_B_ *T*/*q*, the charge‐recombination is bimolecular recombination as dominance, where *k*_B_ is Boltzmann's constant, *T* is temperature, and *q* is the elementary charge. While the slope is closer to 2 *k*_B_ *T*/*q*, the charge‐recombination is monomolecular (trap‐assisted) recombination dominates.^[^ ^60^ ^]^ Thereinto, bimolecular recombination is mainly the recombination of free charges of holes and electrons in the D/A interface, and monomolecular recombination is mostly happened in the defects or trap state for the holes and electrons. As shown in Figure 4f, the slopes of fitted data were 1.45, 1.36, and 1.51 *k*_B_ *T*/*q* for MePBDFCl*_L_*‐, MePBDFCl*_H_*‐, and PBDFCl‐based devices. The result shows that the mesopolymer based devices have fewer trap‐assisted recombination compared to the polymer based devices.

**Figure 5:**
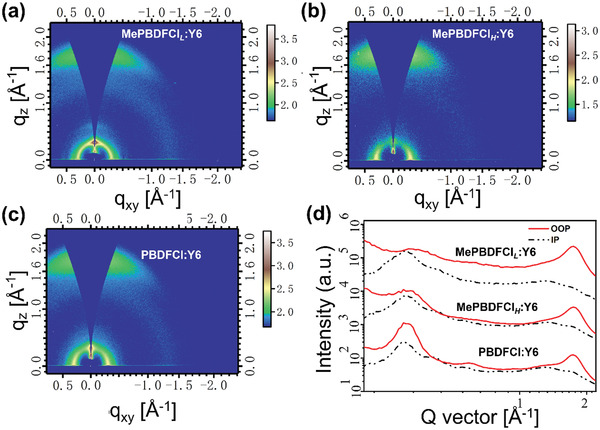
a) The GIWAXS images of MePBDFCl*_L_*: Y6, b) MePBDFCl*_H_*: Y6, and c) PBDFCl: Y6 blend films. d) The corresponding IP and OOP line cuts.^[^ ^16^ ^]^

To meticulously explore the effect of the mesopolymers and polymer on the photovoltaic performance, the details of molecular morphology and orientation features were investigated by GIWAXS for these materials blend films. The images of GIWAXS measurements and the corresponding line cuts of these blend films are exhibited in **Figure** **5**. As shown in Figure 5a–c, all blend films displayed distinctly face‐on molecular orientation in the OOP direction. Meanwhile, the diffraction peaks of all blend films were analogously located at 1.740 Å^–1^ (d‐spacing: 3.610 Å) (Figure 5d). According to the previous research, Y6 as a fullerene‐free acceptor has intense face‐on orientation with a (010) *π*–*π* diffraction at *q* = 1.768 Å^–1^ with relative strong crystallinity.^[^ ^16^ ^]^ The (010) peak of Y6 was undiscovered in these blend films, indicating better miscibility between these donors and Y6. Significantly, MePBDFCl*_L_* and MePBDFCl*_H_* exhibited similar stacking condition with polymer PBDFCl in the blend films, which demonstrated that the mesopolymer had enough potential to form appropriate molecular packing via proper optimization.

**Figure 6:**
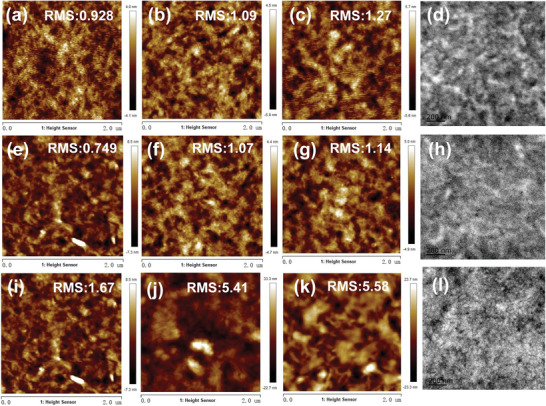
AFM height images (2 × 2 µm) of a–c) MePBDFCl*_L_*: Y6, MePBDFCl*_H_*: Y6, and PBDFCl: Y6 as cast e–g) MePBDFCl*_L_*: Y6, MePBDFCl*_H_*: Y6, and PBDFCl: Y6 with annealing at 110 °C. i–k) MePBDFCl*_L_*: Y6, MePBDFCl*_H_*: Y6, and PBDFCl: Y6 with 0.5% CN and annealing at 110 °C. TEM images d) MePBDFCl*_L_*: Y6 blends with 0.5% CN and annealing at 110 °C, h) MePBDFCl*_H_*: Y6 blends, and l) PBDFCl: Y6 blends with annealing at 110 °C.

Considering that the morphology of the active layer plays an important role for photovoltaic performance, so atomic force microscope (AFM) was performed to investigate the phase separation morphologies of all blend films. As determined by AFM, with the increase of molecular weight, MePBDFCl*_L_*, MePBDFCl*_H_*, and PBDFCl blend films exhibited gradually enhancive root‐mean‐square (RMS) roughness values of 0.928, 1.09, and 1.27 nm, respectively (**Figure** **6**a–c). After the 110 °C annealing process, all blend films exhibited mildly adjusted RMS values (Figure 6e–g). The blend films of MePBDFCl*_H_* and PBDFCl after thermal annealing treatment had more uniform surface without changing their fibrous features. But the MePBDFCl*_L_* blend film exhibited too small RMS (0.749 nm), which was easy to cause exciton recombination, resulting in reduced charge transfer. Under the optimized device fabrication conditions, MePBDFCl*_L_* blend (adding 0.5% CN as additive) displayed the increased phase separation (RMS = 1.67 nm), however, the blend films RMS of MePBDFCl*_H_* and PBDFCl precipitously increased to 5.41 and 5.58 nm, respectively (Figure 6i–k). The excitons might be difficult extraction and could not be efficiently transported due to the serious phase separation and large domains, resulting in reduced *J*_sc_ and FF. The phase images of all blend films showed obvious change process of phase separation in Figure S5, Supporting Information. These results showed the mesopolymer with medium molecular weight could form the analogous phase separation compared with polymer with high molecular weight. The real phase separated morphology of all optimized blends also could be gained by transmission electron microscope. As shown in Figure 6d,h,l, MePBDFCl*_L_* and MePBDFCl*_H_*‐based devices displayed nanoscale phase separation with fiber‐like morphology, which could facilitate charge separation and transport, improving the photovoltaic performance. On the contrary, the PBDFCl‐based devices showed distinctly excessive aggregation. These morphology measurement results and GIWAXS data consistently proved that the BDF‐based mesopolymer could form an effective molecular stacking and crystalline behaviors even in a low molecular weight region (*M*_n_ < 10 kDa), which guarantee enough exciton dissociations and efficient charge transfer.

**Figure 7:**
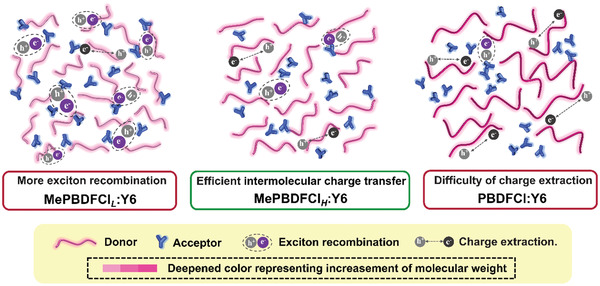
Schematic diagram of the mosopolymers and the polymer and their corresponding active layer microstructures.

To further investigate the miscibility between different donors withY6 acceptor, the contact angles (CA) of two different solvents (water and glycerol) on the donor neat films and Y6 films were measured. The interfacial tensions (*γ*) between the donor and acceptor can be calculated by Wu model.^[^ ^61^ ^]^ As shown in Figure S6, Supporting Information, the water contact angles are 105.3°, 106.0°, and 106.2° for MePBDFCl*_L_*, MePBDFCl*_H_*, and PBDFCl, respectively, and similar trend for glycerol contact angles. The corresponding *γ* values could be calculated, and data is summarized in Table S6, Supporting Information. The Flory‐Huggins interaction parameter *χ* based on the equation of *χ* ∝ ($\sqrt[{}]{\gamma_{A}}-\sqrt[{}]{\gamma_{B}}$)^2^ can evaluate the blend miscibility.^[^ ^62^ ^]^ The calculated *χ* values were 0.0051, 0.0076, and 0.84 for MePBDFCl*_L_*:Y6, MePBDFCl*_H_*:Y6, and PBDFCl:Y6, respectively, which indicated that the MePBDFCl*_L_* and MePBDFCl*_H_* had better miscibility than polymer PBDFCl. The pronounced low miscibility representing large phase separation in the PBDFCl blend films, causing the difficulty in charge extraction (**Figure** **7**).^[^ ^63^, ^64^, ^65^ ^]^ However, the high miscibility for MePBDFCl*_L_* blend film could generate more exciton recombination. The appropriate miscibility in MePBDFCl*_H_*:Y6 could realize efficient intermolecular charge transfer at the donor/acceptor interface.

## 3 Conclusion

In conclusion, high‐performance photovoltaic polymers featuring high molecular weight still have faced some typical challenges such as strong crystallinity leading to low solubility and synthetic difficulty, increasing batch‐to‐batch variations. In this work, the emerging conjugated mesopolymer as donor materials were first constructed to solve these issues. When the new mesopolymer of MePBDFCl*_H_* was designed and synthesized, other two contrastive counterparts of MePBDFCl*_L_* and PBDFCl varying molecular weights were also synthesized for a comprehensive comparison. With the change of molecular weights, these materials exhibited different molecular packing and crystallization behaviors. When blending with acceptor Y6, the MePBDFCl*_L_* with low molecular weight and PBDFCl with high molecular weight showed little or large phase separated morphology, respectively. As a result, the mesopolymer MePBDFCl*_H_* with medium molecular weight exhibited highest PCE of 15.06%, with *V*_oc_ of 0.883 V, *J*_sc_ of 24.96 mA cm^−2^, and FF of 68.32% among the three materials, which was ascribed to the appropriate phase separation and miscibility in the blend film, achieving the efficient intermolecular charge transfer. It is worth mentioning that the mesopolymer is first used in organic photovoltaic and 15.06% is the highest PCE reported for BDF‐based single‐junction OSCs. Additionally, the difference of PCE was less than 5% between MePBDFCl*_L_* and MePBDFCl*_H_*, which indicated that the proposal of mesopolymers was an effective strategy to reduce the batch‐to‐batch variation. This work proves that the BDF unit is a promising electron‐donating core for OSCs. Meanwhile, it also provides an evidence that mesopolymers possess advantages in batch‐to‐batch consistence, good solution‐processability, and high‐performance over OSCs.

## Conflict of Interest

The authors declare no conflict of interest.

## Supporting information

Supporting Information

## Data Availability

The data that supports the findings of this study are available in the supplementary material of this article.
