# Supplementary material for: Conjugated Mesopolymer Achieving 15% Efficiency Single‐Junction Organic Solar Cells
Source: Adv Sci (Weinh). 2022 Jan 22;9(8):2105430. doi: 10.1002/advs.202105430 (PMC8922105; doi:10.1002/advs.202105430)
Supplement: Supplementary file 1 — Supporting Information [file ADVS-9-2105430-s001.pdf]

ADVANCED SCIENCE
Open Access

Supporting Information

for *Adv. Sci.,* DOI: 10.1002/advs.202105430

Conjugated Mesopolymer Achieving 15% Efficiency Single-Junction Organic Solar Cells

*Bing Zheng, Jianling Ni, Shaman Li, Yuchen Yue, Jingxia Wang, Jianqi Zhang, Yongfang Li, Lijun Huo**

Supporting information:

Conjugated Mesopolymer Achieving 15% Efficiency Single-Junction Organic Solar Cells

Bing Zheng,[a] Jianling Ni,[a] Shaman Li,[b] Yuchen Yue,[c] Jingxia Wang,[c] Jianqi Zhang, [d] Yongfang Li, [b] Lijun Huo* [a]

1. Materials and synthesis

The ClBDF-Sn and DTBT-T-BO-Br were purchased from Aldrich or other commercial resources and used as received. Toluene was distilled from sodium benzophenone under nitrogen before using.

Synthesis of PBDF18-Cl

The monomers of ClBDF-Sn (0.0941 g, 0.1mmol) and DTBT-T-BO-Br (0.0907g, 0.1mmol) were dissolved into toluene of 8 mL in a flask under nitrogen. The mixture was flushed with nitrogen for 10 min, and 6 mg of $Pd(PPh_3)_4$ (polymer with high molecular weight need use Pd2(dba)$_3$ and P(o-tolyl)$_3$) was added into the flask. The solution was flushed with nitrogen for an additional 25 min. Then, the reaction mixture was stirred for 24 h (mesopolymer with low molecular weight need control time) at 110 °C. Subsequently the polymer was collected by filtration and purified by washing, extracted on a Soxhlet extractor with methanol and hexane in succession. The final product was obtained by precipitating the chloroform solution in methanol.

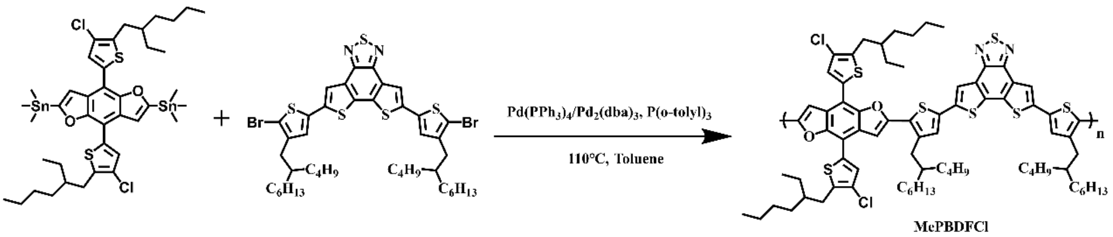

Scheme S1. The synthetic routes of **MePBDFCl**.

2. Characterization of materials

^{1}H NMR spectra were recorded on a Bruker AVANCE 300 MHz spectrometer using $CDCl_3$ as the solvent. The molecular weight of polymers was determined by gel permeation chromatography (GPC) relative to polystyrene standards with chloroform as the eluent. Thermal gravimetric analysis (TGA) was performed on a Perkin-Elmer Pyris 1 thermogravimetric analyzer. UV–vis absorption measurements were carried out on a Hitachi (model U-3010) UV–vis spectrophotometer. Cyclic voltammetric (CV) measurements were carried out in a conventional three-electrode cell using a platinum plate as the working electrode, a platinum wire as the counter electrode, and an Ag/Ag^+ electrode as the reference electrode on a Zahner IM6e Electrochemical workstation in a tetrabutylammonium hexafluorophosphate (Bu_4NPF_6) (0.1 M) acetonitrile solution at a scan rate of 30 mV s^{-1}.

3. Device Fabrication and Characterization

(1) Device Fabrication

An inverted architecture was fabricated with ITO/PEDOT:PSS/active layer/ PDINO/Ag. The ITO-coated glass substrates were sequentially ultrasonicated in soap water, deionized water, acetone, and isopropyl alcohol for at least 15 min, and

ultimately dried in an oven overnight. The ITO-coated glass substrates were treated by uv-ozone for 10 min. Filter the PEDOT:PSS aqueous solution (Baytron P 4083, from HCStarck) through a 0.45 mm filter, and pre-coat it on the pre-cleaned ITO glass at 5000 rpm for 30 seconds, and then heat the ITO substrate in the air at 150°C annealing for 0.5 h. The polymer donor: Y6 (D:A=1:1.2, 16 mg mL^{-1} in total) was dissolved in chloroform (only $MePBDFCl_L$ using 1-chloronaphthalene (CN) (0.5%, v/v) additive). The blended solution was spin-coated on the PEDOT:PSS layer at 3000 rpm for 30s. It is then annealed at 110°C for 10 minutes. Then PDINO methanol solution with a concentration of 0.3 mg mL-1 was deposited on the active layer at a speed of 3000 rpm for 30 seconds to provide a PDINO cathode modification layer. After cooling to room temperature, the sample was transferred to the evaporation chamber. Under the pressure of 1×10^{-5} Pa, about 100 nm of Ag electrode was evaporated and deposited. The device area is 4.0 mm^2. The active area of the devices is 4.0 mm^2. Current density-voltage (*J*-*V*) characteristics were measured by a Keithley 2400 Source Measure Unit, in N_2 atmosphere under an AM 1.5G solar simulator with an irradiation light intensity of $100mw\cdot cm^{-2}$. The external quantum efficiency (EQE) of the devices was measured by using a QEX10 solar cell EQE measurement system (PV measurements.Inc.). The light intensity at each wavelength was calibrated with a standard single-crystal Si photovoltaic cell.

(2) Space-Charge-Limited Current (SCLC)

The current density–voltage (*J*–*V*) characteristics of the hole or electron only devices are fitted by the Mott–Gurney law:

$J = (9/8)\varepsilon_r\varepsilon_0\mu(V^2/L^3)$

where J is the current density, ε_r is the dielectric permittivity of the active layer, ε_0 is the vacuum permittivity, L is the thickness of the active layer, μ is the mobility. $V=V_{app} - V_{bi}$, where V_{app} is the applied voltage, V_{bi} is the offset voltage (V_{bi} is 0 V here). The mobility can be calculated from the slope of the $J^{0.5}$~V curves.

(3) Grazing Incidence Wide-Angle X-ray Scattering (GIWAXS) Characterization

GIWAXS measurements were performed at beamline 7.3.3 at the Advanced Light Source. Samples were prepared on Si substrates using identical blend solutions as those used in devices. The 10 keV X-ray beam was incident at a grazing angle of 0.12°-0.16°, selected to maximize the scattering intensity from the samples. The scattered x-rays were detected using a Dectris Pilatus 2M photon counting detector.

(4) Atomic force microscopy (AFM) Characterization

AFM images were investigated on a Dimension Icon AFM (Bruker) in a tapping mode.

(5) Transmission Electron Microscopy (TEM) Characterization

TEM images were performed on a JEOL JEM-1400 transmission electron microscope. TEM samples were prepared as follows: First, the active layer was spin cast on the top of ITO/PEDOT:PSS substrates; Then, the active layer film was peeled off and floated onto the surface of deionized water; Finally, the floated films were picked up on a carbon film 200 mesh copper grid for TEM measurements.

(6) Contact angle measurements

All the photos were taken by digital camera with macro lens.

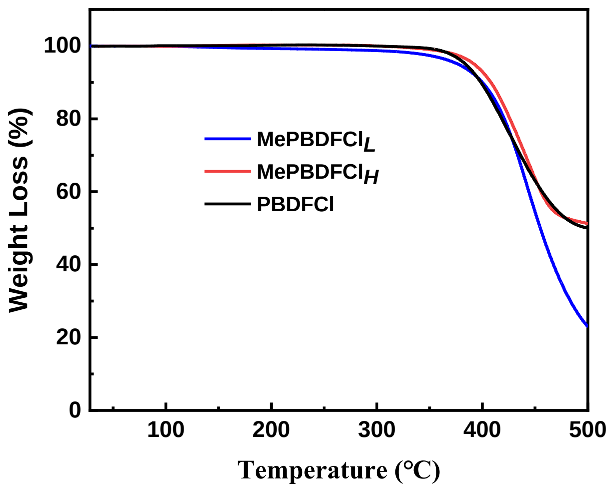

Figure S1. TGA plots of MePBDFCl$_{L}$, MePBDFCl$_{H}$ and PBDFCl with a heating rate of 20 °C/min under the inert atmosphere.

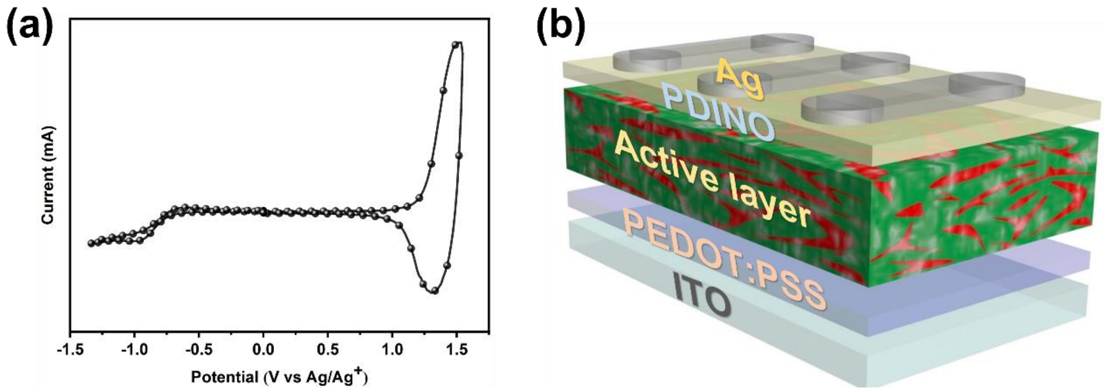

Figure S2. (a) The cyclic voltammetry (CV) curves of the **PBDFCl**. (b) Device structure of the OSCs used in this work.

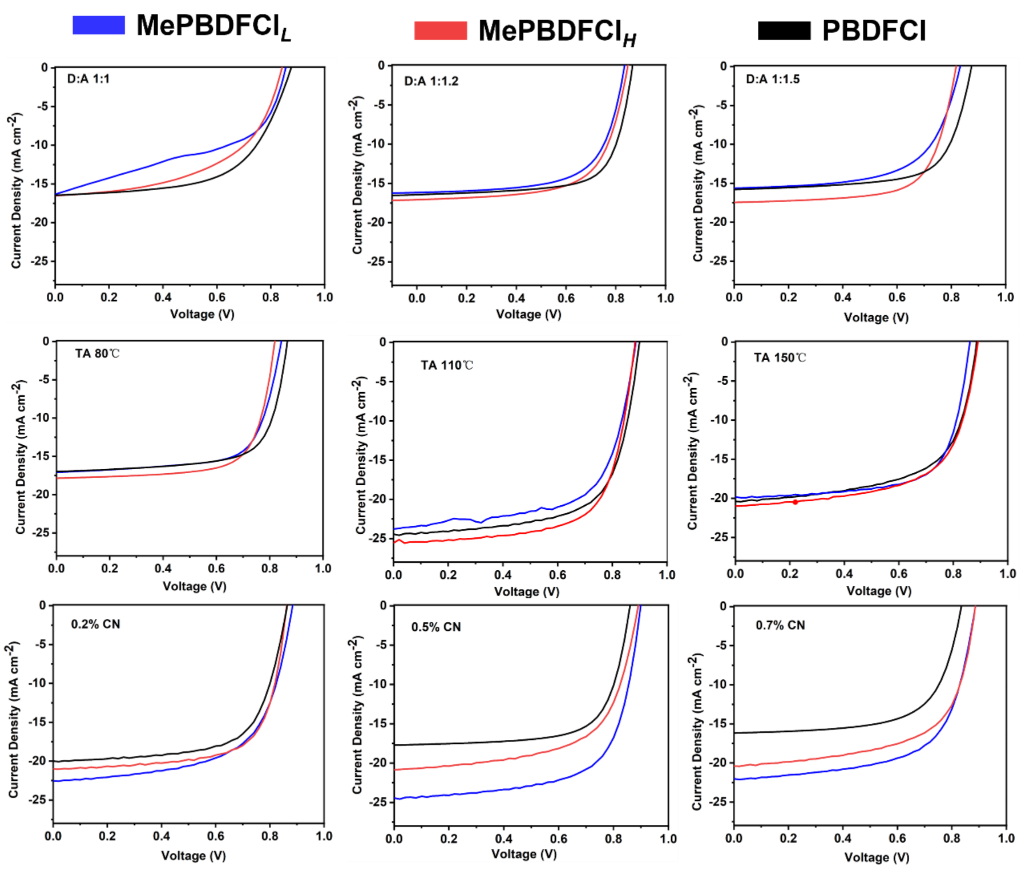

Figure S3. Photovoltaic performance of the OSCs based on MePBDFCl$_L$:Y6, MePBDFCl$_H$:Y6, and PBDFCl:Y6 with different device fabrication conditions.

Table S1. Photovoltaic performance of the OSCs based on MePBDFCl$_L$:Y6, MePBDFCl$_H$:Y6, and PBDFCl:Y6 with different donor/acceptor ratio, under the illumination of AM 1.5 G, 100 mWcm^{-2}.

D/A Ratio	Concentration (CF)	Polymers:Y6	V_{oc} (V)	J_{sc} (mA/cm^2)	FF (%)	PCE (%)
1:1	16 mg/ml	MePBDFCl$_L$	0.85	16.34	46.19	6.45
		MePBDFCl$_H$	0.84	16.52	53.47	7.43
		PBDFCl	0.86	16.97	68.87	10.09

1:1.2	16 mg/ml	MePBDFCl$_{L}$	0.84	16.16	65.80	8.88
		MePBDFCl$_{H}$	0.85	17.08	65.66	9.51
		PBDFCl	0.87	16.43	70.42	10.03
1:1.5	16 mg/ml	MePBDFCl$_{L}$	0.83	15.63	62.39	8.12
		MePBDFCl$_{H}$	0.82	17.46	68.91	9.84
		PBDFCl	0.84	15.80	68.19	9.10

Table S2. Photovoltaic performance of the OSCs based on MePBDFCl$_{L}$:Y6, MePBDFCl$_{H}$:Y6, and PBDFCl:Y6 with different annealing temperature, under the illumination of AM 1.5 G, 100 mWcm^{-2}.

Annealing (°C)	D/A Ratio	Polymers:Y6	V_{oc} (V)	J_{sc} (mA/cm^{2})	FF (%)	PCE (%)
80	1:1.2	MePBDFCl$_{L}$	0.84	17.07	69.70	10.03
		MePBDFCl$_{H}$	0.84	17.85	68.82	10.27
		PBDFCl	0.86	16.99	70.54	10.37
110	1:1.2	MePBDFCl$_{L}$	0.88	23.77	64.57	13.59
		MePBDFCl$_{H}$	0.88	24.96	68.32	15.06
		PBDFCl	0.86	24.70	62.52	13.28
150	1:1.2	MePBDFCl$_{L}$	0.84	19.84	64.74	10.83
		MePBDFCl$_{H}$	0.86	21.01	61.76	11.22
		PBDFCl	0.86	20.39	64.21	11.26

Table S3. Photovoltaic performance of the OSCs based on MePBDFCl$_L$:Y6, MePBDFCl$_H$:Y6, and PBDFCl:Y6 with different additive content, under the illumination of AM 1.5 G, 100 mWcm^{-2}.

Additive (CN) (%)	Annealing (°C)	D/A Ratio	Polymers:Y6	V_{oc} (V)	J_{sc} (mA/cm^2)	FF (%)	PCE (%)
0.2	110	1:1.2	MePBDFCl$_L$	0.88	22.53	62.58	12.41
			MePBDFCl$_H$	0.86	21.02	61.76	11.16
			PBDFCl	0.86	20.00	60.77	10.45
0.5	110	1:1.2	MePBDFCl$_L$	0.89	24.21	66.63	14.35
			MePBDFCl$_H$	0.88	20.84	51.67	9.48
			PBDFCl	0.86	17.97	55.61	8.59
0.7	110	1:1.2	MePBDFCl$_L$	0.88	22.04	57.82	11.21
			MePBDFCl$_H$	0.88	20.39	51.28	9.20
			PBDFCl	0.84	16.43	53.58	7.39

Table S4. The corresponding photovoltaic performance of BDF-based materials in recent years.

No.	Acceptor/donor	J_{sc} (mA cm^{-2})	V_{oc} (V)	FF (%)	PCE	ref
1	PBDFDTBT/PC$_{71}$BM	11.77	0.78	54.6	5.01	1
2	PBDF/PCBM	3.45	0.50	33.0	0.57	2
3	*syn*-PBDFID/PC$_{71}$BM	2.89	0.85	59.0	1.44	3
4	PBDFNTDO/PC$_{71}$BM	9.14	0.87	59.2	4.71	4

5	PBDFTT-C/$PC_{71}BM$	10.45	0.66	63.8	4.40	5
6	PBDFTT-CF/$PC_{71}BM$	13.88	0.63	59.8	5.23	6
7	PBDFTPD/$PC_{71}BM$	11.20	0.97	68.0	7.40	7
8	PBDFNBO/$PC_{71}BM$	9.90	0.93	53.0	5.00	8
9	$P_{in}BDFID$/PCBM	1.66	0.73	48.6	0.59	9
10	P1/$PC_{71}BM$	7.00	0.69	60.0	2.89	10
11	PBDFDODTBT:$PC_{71}BM$	9.87	0.69	65.3	4.45	11
12	PFBT-T/$PC_{71}BM$	12.49	0.77	60.4	5.83	12
13	PBDFFBT/$PC_{71}BM$	9.17	0.62	58.4	3.30	13
14	PBDFs-DTBTff/$PC_{71}BM$	11.04	0.76	65.8	5.48	14
15	P1/PCBM	3.71	0.83	39.0	1.19	15
16	PBDFTTPD/$PC_{71}BM$	4.40	0.90	58.6	2.32	16
17	PBDFTz-SBP/ITIC	18.56	0.89	75.1	12.42	17
18	PBDF-T1/$PC_{71}BM$	13.28	0.92	77.4	9.43	18
19	P(BDF-FDPP)/$PC_{71}BM$	11.18	0.67	72.0	5.40	19
20	P3HT:PBDO-T-TDP/PCBM	9.50	0.58	56.4	3.10	20
21	P9/$PC_{71}BM$	9.81	0.80	58.7	4.61	21
22	PBDFTT-CF-T/$PC_{71}BM$	13.04	0.78	61.5	6.26	22
23	PBDFT-BT/$PC_{71}BM$	9.94	0.73	60.9	4.42	23
24	P4/$PC_{71}BM$	12.11	0.65	64.0	5.23	24
25	PBDFF–Bz/*m*-ITIC	16.57	0.94	66.0	10.28	25
26	PBDFF-FBz/*m*-ITIC	14.43	0.82	69.4	8.79	26
27	J81/*m*-ITIC	16.48	0.96	69.8	11.05	27
28	P2/$PC_{71}BM$	8.74	0.83	61.2	4.44	28

29	PBDFS-fBz/ITIC	15.26	0.88	67.0	9.00	29
30	L2/TTPT-T-4F	22.17	0.86	73.6	14.0	30
31	F11/*m*-ITIC	17.75	0.92	69.6	11.37	31
32	PBDFPBz/IT-M	18.27	1.02	69.3	12.93	32
33	F13/Y6	22.61	0.80	70.2	12.71	33
34	DCA3TBDF/$PC_{61}BM$	7.32	0.90	67.9	4.49	34
35	M2/$PC_{61}BM$	11.40	0.80	60.0	5.50	35
36	BDF2/$PC_{61}BM$	4.25	0.60	50.0	1.65	36
37	B1/$PC_{61}BM$	11.10	0.82	55.5	5.00	37

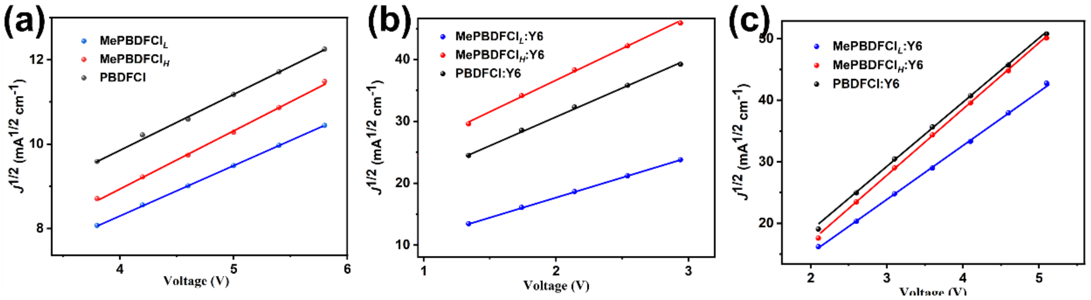

Figure S4. J-V characteristics of a) hole-only devices based on MePBDFCl$_L$, MePBDFCl$_H$, and PBDFCl neat films, b) hole-only devices based on the MePBDFCl$_L$:Y6, MePBDFCl$_H$:Y6, and PBDFCl:Y6 blend films, c) electron-only devices based on the MePBDFCl$_L$:Y6, MePBDFCl$_H$:Y6, and PBDFCl:Y6 blend films.

Table S5. The hole and electron mobility and the ratio μ_h/μ_e of neat and blend films.

Active layer	μ_h (cm^2 V^{-1} s^{-1})	μ_e (cm^2 V^{-1} s^{-1})	μ_h/μ_e
MePBDFCl$_L$	3.49×10^{-4}		

MePBDFCl$_H$	4.78×10^{-4}		
PBDFCl	4.46 ×10^{-4}		
MePBDFCl$_L$:Y6	3.17 × 10^{-4}	3.56 × 10^{-4}	0.89
MePBDFCl$_H$:Y6	4.57 × 10^{-4}	4.84 × 10^{-4}	0.94
PBDFCl:Y6	3.73 × 10^{-4}	4.45× 10^{-4}	0.84

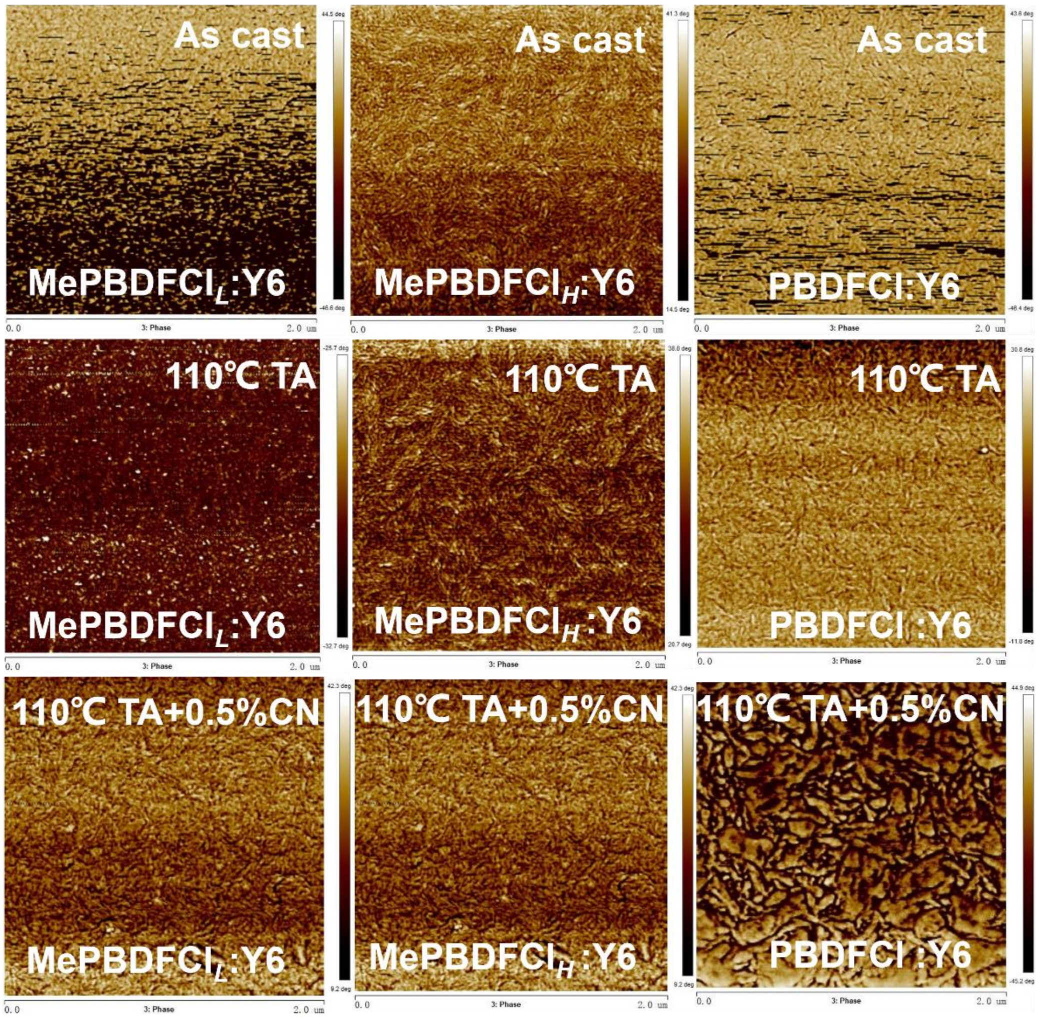

Figure S5. AFM phase images (2 × 2 μm) of (a-c) MePBDFCl$_L$:Y6, MePBDFCl$_H$:Y6, and PBDFCl:Y6 as cast (d-f) MePBDFCl$_L$:Y6, MePBDFCl$_H$:Y6, and PBDFCl:Y6 with annealing at 110 °C. (g-i) MePBDFCl$_L$:Y6, MePBDFCl$_H$:Y6, and PBDFCl:Y6 with 0.5% CN and annealing at 110 °C.

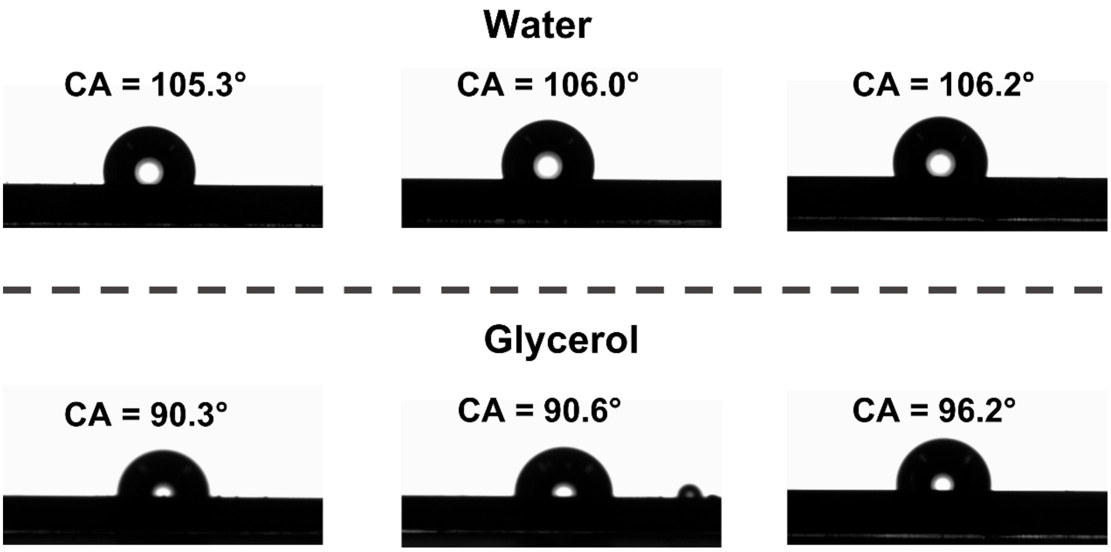

Figure S6. Contact angle images of water and glycerol on $MePBDFCl_L$, $MePBDFCl_H$, and PBDFCl films.

Table S6. Contact angle of water and glycerol and surface tension of $MePBDFCl_L$, $MePBDFCl_H$, PBDFCl and Y6 films.

Neat film	θ_{water} [°]	$\theta_{glycerol}$ [°]	γ [mN m^{-1}]	χ
$MePBDFCl_L$	105.3	90.3	20.91	0.0051
$MePBDFCl_H$	106.0	90.6	20.77	0.0076
PBDFCl	106.2	96.2	13.91	0.84
Y6	90.4	81	21.78	

References

[1] L. Huo, Y. Huang, B. Fan, X. Guo, Y. Jing, M. Zhang, Y. Li, J. Hou, *Chem Commun* **2012**, *48*, 3318.

[2] H. Li, P. Tang, Y. Zhao, S. X. Liu, Y. Aeschi, L. Deng, J. Braun, B. Zhao, Y. Liu, S. Tan, W. Meier, S. Decurtins, *Journal of Polymer Science Part A: Polymer Chemistry* **2012**, *50*, 2935.

[3] C. Hu, Y. Fu, S. Li, Z. Xie, Q. Zhang, *Polymer Chemistry* **2012**, *3*, 2949.

[4] X. Chen, B. Liu, Y. Zou, L. Xiao, X. Guo, Y. He, Y. Li, *Journal of Materials Chemistry* **2012**, *22*, 17724.

[5] B. Liu, X. Chen, Y. Zou, Y. He, L. Xiao, X. Xu, L. Li, Y. Li, *Polym. Chem.* **2013**, *4*, 470.

[6] L. Huo, Z. Li, X. Guo, Y. Wu, M. Zhang, L. Ye, S. Zhang, J. Hou, *Polymer Chemistry* **2013**, *4*, 3047.

[7] J. Warnan, C. Cabanetos, A. El Labban, M. R. Hansen, C. Tassone, M. F. Toney, P. M. Beaujuge, *Adv Mater* **2014**, *26*, 4357.

[8] B. Liu, L. Chen, X. Wang, L. Lic, G. Wang, *New J. Chem.* **2021**, *45*, 2710.

[9] B. M. Kobilka, A. V. Dubrovskiy, M. D. Ewan, A. L. Tomlinson, R. C. Larock, S. Chaudhary, E. M. Jeffries, *Chem Commun* **2012**, *48*, 8919.

[10] B. M. Kobilka, B. J. Hale, M. D. Ewan, A. V. Dubrovskiy, T. L. Nelson, V. Duzhko, M. Jeffries-El, *Polymer Chemistry* **2013**, *4*, 5329.

[11] B. Liu, X. Chen, Y. Zou, L. Xiao, X. Xu, Y. He, L. Li, Y. Li, *Macromolecules* **2012**, *45*, 6898.

[12] T. Lei, W. Song, B. Fanady, T. Yan, L. Wu, W. Zhang, L. Xie, L. Hong, Z. Ge, *Polymer* **2019**, *172*, 391.

[13] L. Xiao, B. Liu, X. Chen, Y. Li, W. Tang, Y. Zou, *RSC Advances* **2013**, *3*, 11869.

[14] J. Yu, G. Luo, L. Bian, B. Zhou, X. Yin, Z. Xu, F. Zhang, P. Deng, H. Wu, W. Tang, *Dyes and Pigments* **2016**, *131*, 356.

[15] P. Sista, P. Huang, S. S. Gunathilake, M. P. Bhatt, R. S. Kularatne, M. C. Stefan, M. C. Biewer, *Journal of Polymer Science Part A: Polymer Chemistry* **2012**, *50*, 4316.

[16] W. Liu, D. He, B. Qiu, L. Jiang, G. Chen, H. Peng, Y. Zou, *Journal of Macromolecular Science, Part A: Pure and Applied Chemistry* **2015**, *52*, 752.

[17] S. Qiao, X. Li, H. Wang, B. Zhang, Z. Li, J. Zhao, W. Chen, R. Yang, *Solar RRL* **2019**, *3*, 1900159.

[18] L. Huo, T. Liu, B. Fan, Z. Zhao, X. Sun, D. Wei, M. Yu, Y. Liu, Y. Sun, *Adv Mater* **2015**, *27*, 6969.

[19] J. Du, A. Fortney, K. E. Washington, M. C. Biewer, T. Kowalewski, M. C. Stefan, *Journal of Materials Chemistry A* **2017**, *5*, 15591.

[20] M. G. Murali, A. D. Rao, S. Yadav, P. C. Ramamurthy, *Polymer Chemistry* **2015**, *6*, 962.

[21] L. Bian, J. Hai, E. Zhu, J. Yu, Y. Liu, J. Zhou, G. Ge, W. Tang, *Journal of Materials Chemistry A* **2015**, *3*, 1920.

[22] L. Huo, L. Ye, Y. Wu, Z. Li, X. Guo, M. Zhang, S. Zhang, J. Hou, *Macromolecules* **2012**, *45*, 6923.

[23] Y. Zhang, L. Gao, C. He, Q. Sun, Y. Li, *Polym. Chem.* **2013**, *4*, 1474.

[24] P. Huang, J. Du, S. S. Gunathilake, E. A. Rainbolt, J. W. Murphy, K. T. Black, D. Barrera, J. W. P. Hsu, B. E. Gnade, M. C. Stefan, M. C. Biewer, *Journal of*

Materials Chemistry A **2015**, *3*, 6980.

[25] Y. Gao, Z. Wang, J. Zhang, H. Zhang, K. Lu, F. Guo, Y. Yang, L. Zhao, Z. Wei, Y. Zhang, *Journal of Materials Chemistry A* **2018**, *6*, 4023.

[26] R. Zhu, Z. Wang, Y. Gao, Z. Zheng, F. Guo, S. Gao, K. Lu, L. Zhao, Y. Zhang, *Macromol Rapid Commun* **2019**, *40*, 1900227.

[27] H. Bin, L. Zhong, Y. Yang, L. Gao, H. Huang, C. Sun, X. Li, L. Xue, Z.-G. Zhang, Z. Zhang, Y. Li, *Advanced Energy Materials* **2017**, *7*, 1700746.

[28] Z. Cong, B. Zhao, H. Wu, Z. Guo, W. Wang, G. Luo, J. Xu, Y. Xia, C. Gao, Z. An, *Polymer* **2015**, *67*, 55.

[29] Y. Gao, Z. Wang, J. Zhang, H. Zhang, K. Lu, F. Guo, Z. Wei, Y. Yang, L. Zhao, Y. Zhang, *Macromolecules* **2018**, *51*, 2498.

[30] X. Li, K. Weng, H. S. Ryu, J. Guo, X. Zhang, T. Xia, H. Fu, D. Wei, J. Min, Y. Zhang, H. Y. Woo, Y. Sun, *Advanced Functional Materials* **2019**, *30*, 1906809.

[31] E. He, Y. Lu, Z. Zheng, F. Guo, S. Gao, L. Zhao, Y. Zhang, *Journal of Materials Chemistry C* **2020**, *8*, 139.

[32] Y. Gao, Z. Shen, F. Tan, G. Yue, R. Liu, Z. Wang, S. Qu, Z. Wang, W. Zhang, *Nano Energy* **2020**, *76*, 104964.

[33] E. He, Z. Zheng, Y. Lu, F. Guo, S. Gao, X. Pang, G. T. Mola, L. Zhao, Y. Zhang, *Journal of Materials Chemistry A* **2020**, *8*, 11381.

[34] Z. Du, Y. Chen, W. Chen, S. Qiao, S. Wen, Q. Liu, D. Zhu, M. Sun, R. Yang, *Chem Asian J* **2014**, *9*, 2621.

[35] D. Qian, B. Liu, S. Wang, S. Himmelberger, M. Linares, M. Vagin, C. Müller, Z.

Ma, S. Fabiano, M. Berggren, A. Salleo, O. Inganäs, Y. Zou, F. Zhang, *Journal of Materials Chemistry A* **2015**, *3*, 24349.

[36] A. Faurie, F. Gohier, P. Frère, *Dyes and Pigments* **2018**, *154*, 38.

[37] B. Liu, X. Wang, L. Li, G. Wang, *RSC Advances* **2020**, *10*, 39916.
